# Outbreak of *Salmonella enterica* Serovar Reading Linked to Dried Bovine Meat, New South Wales, Australia, 2023

**DOI:** 10.3201/eid3208.251454

**Published:** 2026-08

**Authors:** Elenor Kerr, Sophie E. Dwyer, David S. Kennedy, Vendula Blaya-Novakova, Anne McIntosh, Patrick Peacock, Basel Suliman, Grace A. Blackwell, Qinning Wang, Vitali Sintchenko, Kirsty Hope, Isabel Hess

**Affiliations:** Sydney Local Health District Public Health Unit, Sydney, New South Wales, Australia (E. Kerr, S.E. Dwyer, D.S. Kennedy, V. Blaya-Novakova, I. Hess); NSW Department of Primary Industries and Regional Development, Sydney (A. McIntosh); National Centre for Epidemiology and Population Health, Australian National University, Canberra, Australian Capital Territory, Australia (P. Peacock); Centre for Infectious Diseases and Microbiology Laboratory Services, Institute of Clinical Pathology and Medical Research, NSW Health Pathology, Sydney (P. Peacock, B. Suliman, G. Blackwell, Q. Wang, V. Sintchenko); Health Protection NSW, Sydney (K. Hope)

**Keywords:** bacteria, disease outbreaks, foodborne diseases, food safety, microbiology, humans, meat products, *Salmonella enterica*, food poisoning, whole-genome sequencing, Australia

## Abstract

An outbreak of *Salmonella* *enterica* serovar Reading in Sydney, New South Wales, Australia, was linked to a restaurant and meat products purchased from South Asian grocery stores. *Salmonella* Reading was isolated from dried bovine meat products obtained from the restaurant and traced to an unlicensed meat processor and manufacturer of dried meat products.

Sporadic outbreaks of *Salmonella enterica* serovar Reading have been linked to commercial turkey production (*1*) and fresh produce (*2*). In Australia, *Salmonella* Reading is uncommon; an average of 117 notifications occurred per year during 2017–2022 (range 102–137 notifications) (*3*). In August 2023, an above-average number of *Salmonella* Reading laboratory cases were identified in New South Wales (NSW); 23 were reported in July 2023, compared with a monthly average of 2 (range 0–7). Early interviews identified that most case-patients shared the same cultural background and reported dining at restaurant A or consuming a dried meat product purchased from South Asian grocery stores. A concurrent food safety complaint by a patron against restaurant A prompted an urgent inspection by the NSW Department of Primary Industries and Regional Development (DPIRD) and a formal investigation by the Sydney Local Health District Public Health Unit. This article describes investigations undertaken to control this outbreak with the goal of raising awareness of *Salmonella* transmission risks from dried meats. Human research ethics approval was not required for this study because it was conducted as a public health investigation under the NSW Public Health Act 2010.

## The Study

**Figure 1:**
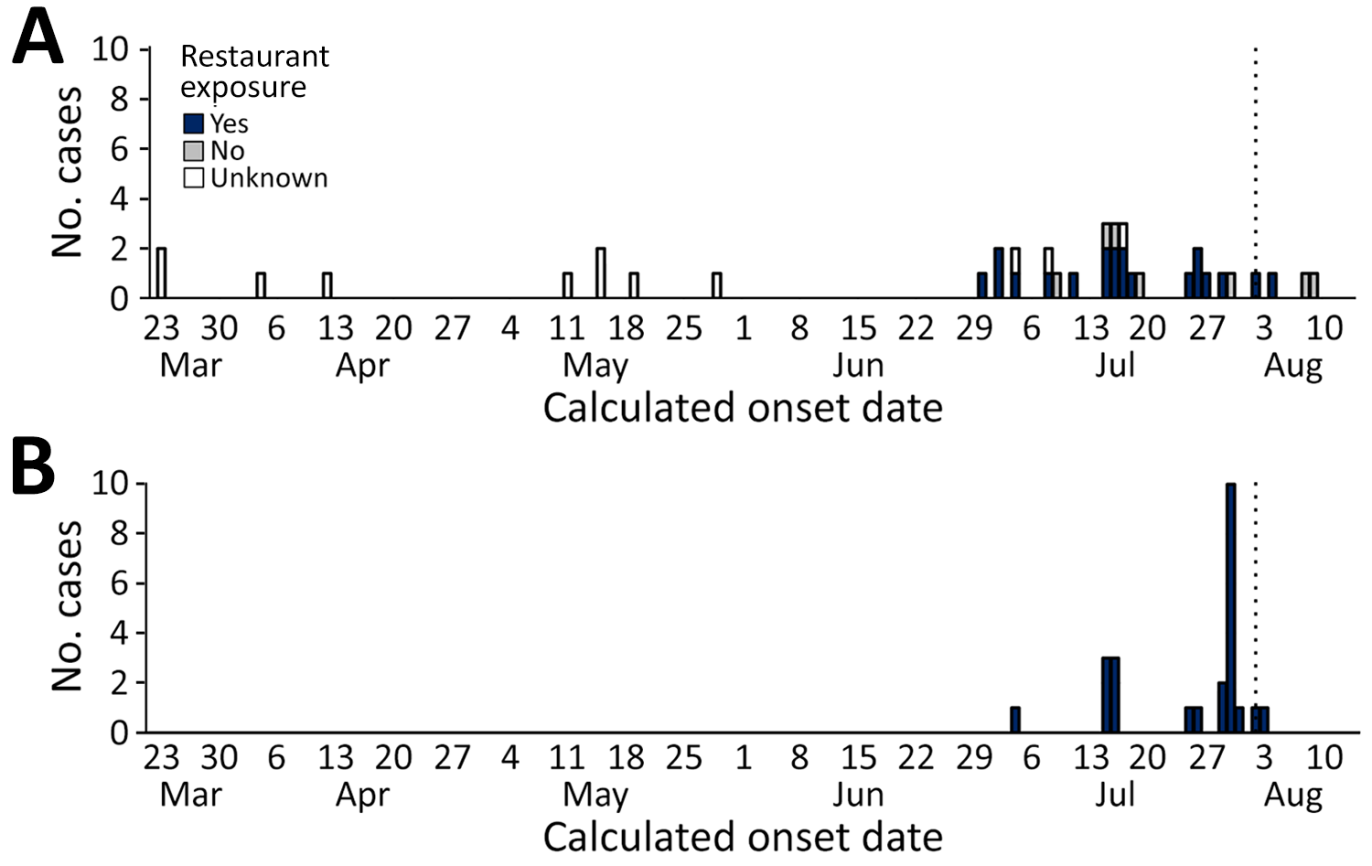
Number of *Salmonella enterica* serovar Reading cases by calculated onset date, case category, and restaurant exposure in study of outbreak of *Salmonella* Reading linked to dried bovine meat, Sydney, New South Wales, Australia, March 23–August 14, 2023. A) Confirmed cases; B) possible cases. Dotted lines indicate date of restaurant closure.

We identified 63 outbreak cases in NSW (39 confirmed, 24 possible) (Table 1; Appendix). Cases were sporadically reported during March–June (range 0–2 cases per month), followed by a substantial increase in July (Figure 1). Confirmed case-patients were 1–57 (median 27.5) years of age, and all with known ethnicity were from the same South Asia country (37/37; 100%).

**Table 1:** Summary of case numbers by clinical status and food exposures in study of outbreak of *Salmonella enterica* serovar Reading linked to dried bovine meat, Sydney, New South Wales, Australia, 2023*

Case category	No. cases	No. (%) patients				
		Sought care at ED†	Hospitalized	Ate at restaurant A	Non–restaurant attendees who consumed dried bovine meat	Non–restaurant attendees who consumed fresh goat meat
Confirmed	39	20/31 (65)	8/31(26)	20/27‡ (74)	4/7 (57)	3/7 (43)
Possible	24	0/4	0/4	24/24 (100)	NA	NA
Total	63	20/35 (57)	8/35 (23)	44/51 (86)	4/7 (57)	3/7 (43)

*Data source: *Salmonella* Reading outbreak line list, Health Protection NSW, Sydney. Extracted on June 5, 2025. ED, emergency department; NA, not applicable. †Emergency department and hospitalization status data were available for 35 cases. ‡Food exposure data for the 7 d before illness onset were available for 27 interviewed confirmed outbreak cases. Restaurant exposure data was available for all possible cases.

Twenty case-patients sought care at an emergency department, and 8 were hospitalized (Table 1). *Salmonella* hypothesis-generating questionnaires were used to collect information from confirmed case-patients. Restaurant-exposed case-patients completed a menu-based questionnaire for restaurant A. Telephone interviews were completed for 51 cases (51/63; 81%). We identified 9 laboratory-confirmed cases retrospectively through whole-genome sequencing (WGS) several months after illness onset, when interviewing was not feasible because reliable food recall was unlikely; 3 patients were lost to follow-up.

Most outbreak cases were linked to recent consumption of food from restaurant A (44 cases [20 laboratory-confirmed, 24 possible]). The median time from food consumption to symptom onset was 1 day (range 0–3 days). Seven persons with laboratory-confirmed infection did not eat at restaurant A; 6 of those persons reported consuming fresh or dried meat purchased from South Asian grocery stores before illness onset.

We conducted a case–control analysis of menu items from restaurant A. No customer reservation data were available, so we recruited controls through snowball sampling from laboratory-confirmed cases. We defined a dining party as patrons seated together under 1 booking. We calculated attack rates as the number of cases divided by the number of diners. We estimated crude odds ratios with 95% CIs using logistic regression models fitted with Firth’s penalized maximum-likelihood (https://doi.org/10.32614/CRAN.package.logistf), which reduces small-sample and separation bias by adding a penalization term to the likelihood. The analysis included 62 persons (41 case-patients and 21 controls) across 15 dining groups; attack rates by group ranged from 20% (1/5) to 100% (4/4). Four items (mattar paneer, chicken choila, achar, and goat Bhutan) were significantly associated with illness (crude odds ratio >1; p<0.05) (Table 2).

**Table 2:** Summary of food/menu items reportedly consumed by persons at restaurant A during outbreak of *Salmonella enterica* serovar Reading linked to dried bovine meat, Sydney, New South Wales, Australia, June 26–July 29, 2023*

Food or menu item	Case-patients	Controls	Crude odds ratio†	
			Point estimate (95% CI)	p value
Mattar paneer‡	12/13 (92)	26/45 (58)	6.13 (1.30–59.66)	0.019
Sadeako bhatmas [beans]	4/4 (100)	34/54 (63)	5.35 (0.53–722.41)	0.18
Newari chicken choila khaja set	4/4 (100)	34/54 (63)	5.35 (0.53–722.41)	0.18
Chicken choela [choila]‡	15/17 (88)	23/41 (56)	4.88 (1.28–27.00)	0.018
Achar‡	14/16 (88)	24/42 (57)	4.38 (1.14–24.28)	0.03
Garlic naan	3/3 (100)	35/55 (64)	4.04 (0.36–553.92)	0.291
Goat Bhutan‡	16/19 (84)	22/39 (56)	3.67 (1.07–15.77)	0.038
Green salad [carrot and cucumber]	11/13 (85)	27/45 (60)	3.10 (0.79–17.34)	0.11
Sayabhale chicken	2/2 (100)	36/56 (64)	2.81 (0.21–394.67)	0.469
Soft drink [cola]	9/11 (82)	29/47 (62)	2.38 (0.59–13.53)	0.235
Vegetable pakora [Pyaji/pyaj pakoda]	12/15 (80)	26/43 (60)	2.36 (0.67–10.27)	0.187
Tomato pickles	11/14 (79)	27/44 (61)	2.09 (0.59–9.15)	0.263
Goat/mutton curry	18/24 (75)	20/34 (59)	2.01 (0.68–6.47)	0.213
Beer	4/5 (80)	34/53 (64)	1.70 (0.29–17.80)	0.579
Papadum [Papad]	14/19 (74)	24/39 (62)	1.67 (0.54–5.73)	0.384
Newari Bhutan khaja set	1/1 (100)	37/57 (65)	1.64 (0.08–243.81)	0.757
Chicken momo mutton soup	1/1 (100)	37/57 (65)	1.64 (0.08–243.81)	0.757
Potato, cucumber, and carrot pickle [Aloo ko achar]	6/8 (75)	32/50 (64)	1.48 (0.34–8.71)	0.617
Steamed basmati rice	19/27 (70)	19/31 (61)	1.47 (0.51–4.41)	0.48
Chili chicken	3/4 (75)	35/54 (65)	1.28 (0.19–13.91)	0.804
Cocktails	13/19 (68)	25/39 (64)	1.18 (0.39–3.84)	0.773
Furandana [fried beaten rice]	8/12 (67)	30/46 (65)	1.02 (0.29–4.01)	0.974
Gulab Jamun [lalmohan]	14/22 (64)	24/36 (67)	0.87 (0.29–2.63)	0.802
Macha [Fish] Thakali Set	5/8 (63)	33/50 (66)	0.82 (0.19–3.90)	0.792
Pressure cooker momo in goat bone soup	10/17 (59)	28/41 (68)	0.66 (0.21–2.12)	0.481
Plain lassi	1/2 (50)	37/56 (66)	0.52 (0.04–6.72)	0.585
Mango lassi	1/2 (50)	37/56 (66)	0.52 (0.04–6.72)	0.585
Pressure cooker vegetarian momo [bamboo momo]	1/2 (50)	37/56 (66)	0.52 (0.04–6.72)	0.585
Roast potato with Sichuan pepper	3/6 (50)	35/52 (67)	0.49 (0.10–2.55)	0.386
Sukuti [buff or mutton]	5/10 (50)	33/48 (69)	0.46 (0.12–1.78)	0.257
Jwai Thakali Set	2/5 (40)	36/53 (68)	0.34 (0.05–1.92)	0.219
Chauchau chapatey	1/3 (33)	37/55 (67)	0.30 (0.03–2.39)	0.246
Momo [buff, boar, chicken]	4/11 (36)	34/47 (72)	0.23 (0.06–0.86)	0.029
Chicken lollipop	1/4 (25)	37/54 (69)	0.20 (0.02–1.32)	0.095
Fried game bird/pheasant [Kalij thali]	1/4 (25)	37/54 (69)	0.20 (0.02–1.32)	0.095
Chicken sausages with tomato and chili	0/2 (0)	38/56 (68)	0.10 (0.00–1.26)	0.076

*Values are no. (%) except as indicated. Data source: *Salmonella *reading outbreak line list, Health Protection New South Wales, Sydney. Extracted on June 5, 2025. Because of incomplete data, 1 dining group from August 1 is not included in the analysis for food items consumed. Items only consumed as part of takeaway by the foodborne illness complaint group were excluded from the analysis. †Firth logistic regression used to estimate crude odds ratios due to incomplete separation for food items.  ‡Considered statistically significant with a crude odds ratio >1 and p value <0.05.

Inspection of restaurant A found inadequate dishwasher temperature, evidence of rodents, improper food storage temperatures, and unsafe thawing practices (Appendix). Of 58 samples collected, 25 (43%) were positive for *Salmonella* Reading, including samples from kitchen surfaces, cleaning implements, hot and cold menu items (chicken and duck choila, chilli chicken, and chicken chow mein), and 2 sealed air-dried bovine meat samples. Environmental samples were highly genomically related to human isolates (0–5 single-nucleotide polymorphisms).

Detection of *Salmonella* Reading in sealed dried meat packages from restaurant A supported the hypothesis that this was a source of *Salmonella*. Multiple positive food and environmental samples indicated poor hygiene and food-handling practices, likely causing cross-contamination and amplifying the outbreak. Cross-contamination is further supported by detections in dishes without bovine products, including 1 of the 4 suspected menu items identified in the case–control analysis. Previous salmonellosis outbreaks indicate that contamination of numerous dishes from a single source can be encouraged by lapses in restaurant hygiene and food safety (*4*,*5*). Improper temperature control and storage might have contributed to the cross-contamination of dishes.

DPIRD traced ingredients from restaurant A and the implicated grocery stores. After *Salmonella* Reading was detected in sealed, dried meat from restaurant A, DPIRD investigated the manufacturer (manufacturer B) and found unsuitable facilities, no verification of ingredients, no pH or water activity checks, no controls for the preparation and dehydration processes, and no food safety program. Links to 2 butcher shops (butchers C and D) were identified. *Salmonella* Reading was detected in raw bovine meat from butcher D (the supplier of bovine meat for manufacturer B); however, that isolate was not linked to the outbreak by WGS. Two additional unlicensed food businesses were identified. Enforcement actions were taken against restaurant A and manufacturer B, and manufacturer B issued a product recall (*6*).

Multiple issues with the manufacturing process by the unlicensed manufacturer meant that a safe and suitable product could not be produced (*7*). The long shelf life of the dried bovine meat product might have prolonged the outbreak. Contamination of commercially manufactured and locally produced dried bovine meat products with *Salmonella* spp. has been associated with several international outbreaks (*8*,*9*) because serovars can survive in low-moisture, low–water-activity foods (*10*). A shelf-stable, culturally preferred food product has been implicated in at least 1 other protracted *Salmonella* Reading outbreak among a specific cultural group (*11*). In Australia, dried meat is an atypical source of *Salmonella*, so identifying its consumption among laboratory-confirmed outbreak cases with no exposure to restaurant A was critical to guiding the investigation.

**Figure 2:**
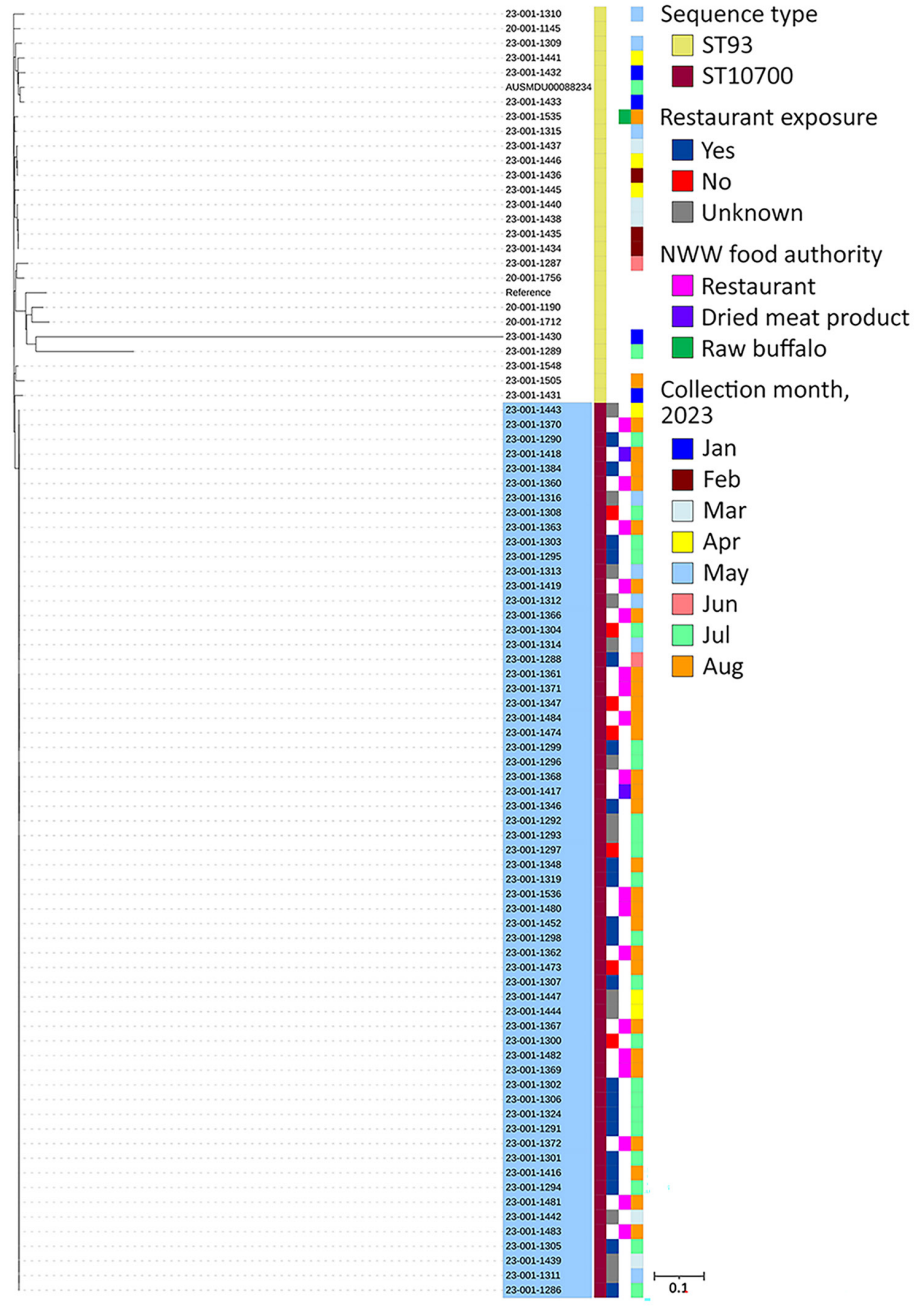
Phylogenetic tree of Salmonella Reading genomic surveillance cluster, SalRea-23-0001, including human, food and environmental isolates, in study of outbreak of *Salmonella enterica* serovar Reading linked to dried bovine meat, Sydney, New South Wales, Australia, 2023.

The investigation provided robust microbiological and traceback evidence linking human cases to restaurant contamination, sealed dried bovine product, and fresh/dried meats from grocery stores. Sixty *Salmonella* Reading isolates (41 human isolates from 39 persons and 19 food and environmental isolates) were highly genomically related (0–5 single-nucleotide polymorphism differences) within the genomic surveillance cluster SalRea-23-0001. The genomic cluster was defined as a novel sequence type, 10700 (EnteroBase; https://enterobase.warwick.ac.uk) (Figure 2). We extracted genomic DNA from pure cultures using the QIAGEN DNeasy UltraClean Microbial Kit (https://www.qiagen.com) (Appendix) and submitted the DNA sequence to the National Center for Biotechnology Information Sequence Read Archive (https://www.ncbi.nlm.nih.gov/sra; BioProject accession no. PRJNA489746, SRA sample no. SAMN36942405). 

Limitations of this investigation included incomplete case interviews, possible recall bias in reported food exposures, limited controls, and a small sample size, which might reduce generalizability to other settings. However, timely WGS analysis was critical for linking isolates and confirming traceback findings. Integrating WGS data with demographic and risk factor data validated early hypotheses on sources and at-risk groups. This integrative approach has proven effective for guiding resource allocation in surveillance and outbreak investigations across disease groups (*12*).

## Conclusions

In conclusion, an outbreak of *Salmonella* Reading in NSW, Australia (63 notifications during March–August 2023), was linked to contaminated dried bovine meat products and cross-contamination at restaurant A, amplified by poor hygiene and food-handling practices. Traceback identified an unlicensed manufacturer with inadequate safety controls, prompting enforcement and a product recall. Timely WGS analysis confirmed genomic relatedness among human, food, and environmental isolates and guided subsequent control measures, underscoring the need for strict food safety procedures and integrated genomic surveillance.

Appendix Additional information about outbreak of *Salmonella enterica* serovar Reading linked to dried bovine meat, Sydney, New South Wales, Australia, 2023.
