# Supplementary material for: Outbreak of Salmonella enterica Serovar Reading Linked to Dried Bovine Meat, New South Wales, Australia, 2023
Source: Emerg Infect Dis. 2026 Aug;32(8):1330–4. doi: 10.3201/eid3208.251454 (PMC13426814; doi:10.3201/eid3208.251454)
Supplement: Appendix — Additional information about outbreak of Salmonella enterica serovar Reading linked to dried bovine meat, Sydney, New South Wales, Australia, 2023. [file 25-1454-Techapp-s1.pdf]

Article DOI: https://doi.org/10.3201/eid3208.251454

EID cannot ensure accessibility for supplementary materials supplied by authors. Readers who have difficulty accessing supplementary content should contact the authors for assistance.

Outbreak of *Salmonella enterica* serovar Reading Linked to Dried Bovine Meat, New South Wales, Australia, 2023

Appendix

Case definitions

Case definitions were regularly reviewed and updated based on new information received throughout the investigation (e.g., the identification of cluster cases through sequencing of isolates from March 2023). The final case definitions used are included below.

Confirmed outbreak case – Any notified case of *Salmonella* Reading infection with an illness onset between 23 March and 14 August 2023, which was highly related to other cases by whole genome sequencing (WGS; phylogenetic analysis).

Possible outbreak case – Salmonellosis-like illness in a person with an epidemiologic link to a meal or venue shared by a confirmed or probable outbreak case, in the absence of laboratory evidence of *Salmonella* Reading infection.

Environmental investigation

On the evening of 2 August 2023, NSW DPIRD officers inspected Restaurant A and collected food samples from the kitchen. Officers returned to the restaurant on 3 August 2023 to collect environmental and additional food samples. The restaurant was re-inspected and re-sampled on 9 and 11 August 2023, respectively. The NSW DPIRD conducted a traceback into the source of a variety of ingredients, with a focus on suppliers to the restaurant.

Genomic extraction

The sequencing libraries were prepared using Nextera XT DNA kit (Illumina, Australia), and sequenced using paired-end 150-bp chemistry on the NextSeq 500 instrument (Illumina, Australia). Demultiplexed sequencing reads with >1 × 107 reads per isolate were trimmed based on a minimum quality phred score of 20, and then assembled de novo using SPAdes (version 3.13.0) (*1*). The quality of de novo assemblies was assessed with Quast (version 5.0.2) (*2*).

Phylogenetic analysis

Sequenced raw reads were subjected to an in-house quality control procedure before further analysis and analyzed using the Nullarbor pipeline (v2.0; https://github.com/tseemann/nullarbor). The reads were screened for contaminants using the taxonomic classification tool Centrifuge. The phylogenetic analysis of genomes was performed using single-nucleotide polymorphisms (SNPs), defined as substitutions present in at least 90% of reads, with a minimum coverage depth of 30. The reference genome for the SNP analysis was from the National Centre for Biotechnology Information (NCBI) with nucleotide accession GCA_010762755.2_ASM1076275v2. Core genome SNPs from each sequence were aligned using Snippy-core. The SNP cluster was called based on the phylogenetic tree and SNP distance, and SNPs within <20 were considered likely linked. The maximum-likelihood tree was generated using FastTree 2.1.9 (http://www.microbesonline.org/fasttree).

References

1. Prjibelski A, Antipov D, Meleshko D, Lapidus A, Korobeynikov A. Using SPAdes De Novo Assembler. Curr Protoc Bioinformatics. 2020;70:e102. PubMed https://doi.org/10.1002/cpbi.102

2. Mikheenko A, Prjibelski A, Saveliev V, Antipov D, Gurevich A. Versatile genome assembly evaluation with QUAST-LG. Bioinformatics. 2018;34:i142–50. PubMed https://doi.org/10.1093/bioinformatics/bty266
